# Skin-Targeted Inhibition of PPAR β/δ by Selective Antagonists to Treat PPAR β/δ – Mediated Psoriasis-Like Skin Disease *In Vivo*

**DOI:** 10.1371/journal.pone.0037097

**Published:** 2012-05-14

**Authors:** Katrin Hack, Louise Reilly, Colin Palmer, Kevin D. Read, Suzanne Norval, Robert Kime, Kally Booth, John Foerster

**Affiliations:** 1 Medical Research Institute, College of Medicine, Dentistry, and Nursing, University of Dundee, Dundee, Scotland; 2 Department of Dermatology, College of Medicine, Dentistry, and Nursing, University of Dundee, Dundee, Scotland; 3 Education Division, College of Medicine, Dentistry, and Nursing, University of Dundee, Dundee, Scotland; 4 Medical School Biological Resource Unit, College of Medicine, Dentistry, and Nursing; 5 Biological Chemistry and Drug Discovery Unit, College of Life Sciences, University of Dundee, Dundee, Scotland; University Hospital Hamburg-Eppendorf, Germany

## Abstract

We have previously shown that peroxisome proliferator activating receptor ß/δ (PPAR β/δ is overexpressed in psoriasis. PPAR β/δ is not present in adult epidermis of mice. Targeted expression of PPAR β/δ and activation by a selective synthetic agonist is sufficient to induce an inflammatory skin disease resembling psoriasis. Several signalling pathways dysregulated in psoriasis are replicated in this model, suggesting that PPAR β/δ activation contributes to psoriasis pathogenesis. Thus, inhibition of PPAR β/δ might harbour therapeutical potential. Since PPAR β/δ has pleiotropic functions in metabolism, skin-targeted inhibition offer the potential of reducing systemic adverse effects. Here, we report that three selective PPAR β/δ antagonists, GSK0660, compound 3 h, and GSK3787 can be formulated for topical application to the skin and that their skin concentration can be accurately quantified using ultra-high performance liquid chromatography (UPLC)/mass spectrometry. These antagonists show efficacy in our transgenic mouse model in reducing psoriasis – like changes triggered by activation of PPAR β/δ. PPAR β/δ antagonists GSK0660 and compound 3 do not exhibit systemic drug accumulation after prolonged application to the skin, nor do they induce inflammatory or irritant changes. Significantly, the irreversible PPAR β/δ antagonist (GSK3787) retains efficacy when applied topically only three times per week which could be of practical clinical usefulness. Our data suggest that topical inhibition of PPAR β/δ to treat psoriasis may warrant further exploration.

## Introduction

One prominent clinical aspect of psoriasis is the clinical overlap with metabolic syndrome [1] and its association with increased body mass index [2], indicative of overlapping signalling pathways in psoriasis and other disorders of metabolism and chronic inflammation. Peroxisome proliferator activated receptors (PPAR) beta/delta (PPAR β/δ), one of three PPAR isoforms, is a key regulator of glucose and lipid metabolism [3]. In psoriasis plaques, PPAR β/δ is up-regulated, while the other PPAR isoforms, alpha, and gamma, are down-regulated [4]. PPARs act as regulators of transcription, being activated by lipid ligands to bind cognate cis-acting elements in target promoters upon heterodimerization with retinoic x receptor (RXR) alpha.

In the skin, PPAR β/δ is involved in keratinocyte differentiation and the wound response [5]. It is induced (among other factors) by TNFα [6], [7], stimulates proliferation and blocks apoptosis in keratinocytes [8], and induces angiogenesis [9]. In psoriasis lesions, PPAR β/δ exhibits prominent nuclear localisation in the upper spinous layer [4]. While not expressed in adult inter-follicular skin in mice, its activation in the spinous layer is sufficient to elicit an inflammatory skin disease harbouring major elements of psoriasis. Thus, PPAR β/δ transgenic mice exhibit psoriasis-typical immunological changes, STAT3 activation, as well as psoriasis – specific gene dysregulation [4]. Moreover, the gene dysregulation profile induced by epidermal PPAR β/δ activation significantly overlaps with that characteristic of psoriasis, including faithful replication of well recognised functional clusters such as the entire Il1-module or the cholesterol biosynthesis program, suggesting that the subsets of genes dysregulated by PPAR β/δ activation are also regulated by PPAR β/δ in psoriasis. Collectively, these observations indicate that PPAR β/δ signalling may contribute to the overlap between psoriasis and metabolic, as well as cardiovascular disease [10], since it is up-regulated in chronic inflammation and regulated by caloric intake [11], [12]. TNFα, obesity, chronic inflammation, and dyslipidemia all may increase the penetrance of psoriasis by inducing PPAR β/δ expression and/or activation. Taken together, several lines of evidence suggest that PPAR β/δ activation contributes to psoriasis pathogenesis and that blocking its activation may reduce disease activity.

In light of the complex role PPAR β/δ exerts in metabolism, a topical ointment approach would seem an attractive targeting strategy in order to minimise the chance of adverse systemic effects. However, isoform – selective PPAR β/δ antagonists have only recently become available [13]–[16], and have not yet been evaluated for their activity *in vivo* via transdermal application. A major limitation in assessing the latter aspect is the availability of a validated and robust method to quantify the active compounds in the skin.

Here we describe the formulation of three selective PPAR β/δ antagonist into ointments and the quantification of their concentration in murine skin. In order to assess their ability to inhibit PPAR β/δ in vivo, we employ a previously described transgenic model [4]. In this model, human PPAR β/δ is constitutively expressed in sebaceous glands. Addition of the PPAR β/δ selective synthetic agonist GW501516 triggers both epidermis-specific transcriptional induction and ligand-mediated activation of PPAR β/δ, causing the development of an inflammatory skin disease with similarity to psoriasis. We show that PPAR β/δ antagonists in ointment formulation can deliver pharmacologically active concentrations in the skin, show very little systemic absorption, that prolonged administration does not cause inflammatory changes, and that they inhibit PPAR β/δ mediated psoriasis-like pathogenesis *in vivo*.

## Results

### Choice of PPAR β/δ selective antagonists

**Figure 1:**
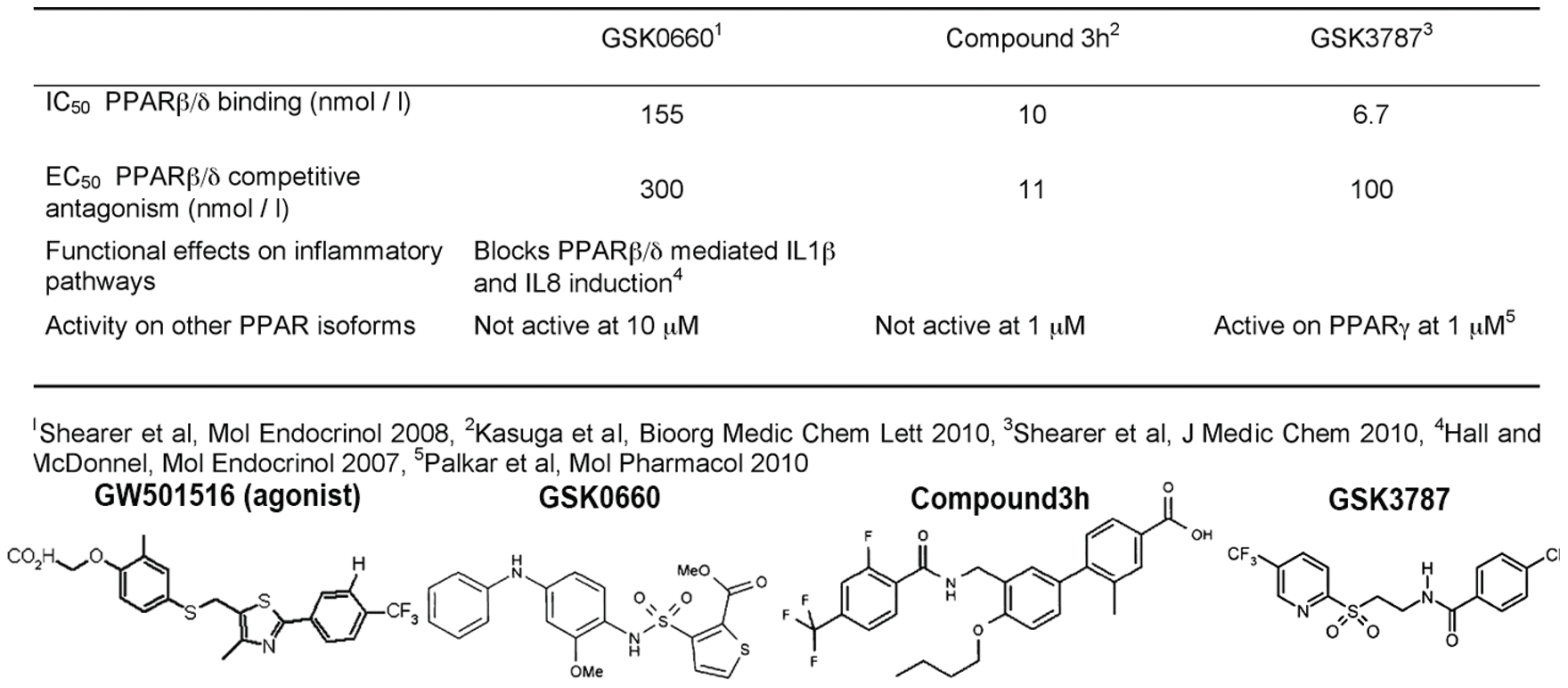
PPAR β/δ selective antagonists used in this study. Chemical structures and in vitro pharmacodynamic data shown are taken from the references listed. The structure of the PPAR β/δ selective agonist GW501516 used in this study is given for comparison.

PPAR β/δ isoform-selective antagonists have only recently been described [13]–[17]. For the present work we used the first one to be reported, GSK0660, based on high antagonist potential, high affinity, its documented anti-inflammatory effect [18], and a reported lack of bioavailability upon systemic administration [17], thus potentially increasing its usefulness as a skin specific targeting compound. In order to ensure that any effects seen *in vivo* are indeed due to PPAR β/δ antagonism and not caused by off-target effects related to the chemical structure of GSK0660, we also included an alternative antagonist, compound 3 h [13], in a subset of experiments. Compound 3 h was chosen because of its low reported Ki (11 nM), high competitive antagonist potency, as well as lack of activity on other PPAR isoforms [13], The structure and key properties of these antagonists are shown in figure 1. An alternative reported compound appears to be less isoform selective [14]. Finally, one additional PPAR β/δ antagonist irreversibly inactivates the receptor by forming a covalent bond (GSK3787) [15], [16]. This compound was included to address the feasibility of achieving treatment effects with less frequent dosing.

### Ointment formulation

A variety of vehicles, additives, and procedures were screened as vehicles for the incorporation for GSK0660 and compound 3 h. These included drug incorporation into liquified vehicle at 70°C, pre-disolvement in DMSO, ethanol, isopropanol, polyethyene glycol 300, and olive oil followed by vehicle incorporation, as well as a variety of vehicles (liquid paraffin, Hydromol ointment, aqueous ointment BP). Drug solubility was assessed using a previously reported method relying on the absence of crystals detectable by polarised microscopy [19]. It was furthermore found that GSK0660 underwent a visible colour change from yellow to green after storage at room temperature and light exposure, indicating instability possibly due to oxidation, which would be predicted given its chemical structure (fig. 1). Thus, alpha-tocopherol was added to GSK0660 preparations to increase stability. The optimised formulation of both compounds is detailed in Methods, was found to be devoid of drug crystals up to 2% (w/w) for both compounds, and exhibited functional activity, as described below.

### Lack of systemic absorption of PPAR β/δ antagonists

**Figure 2:**
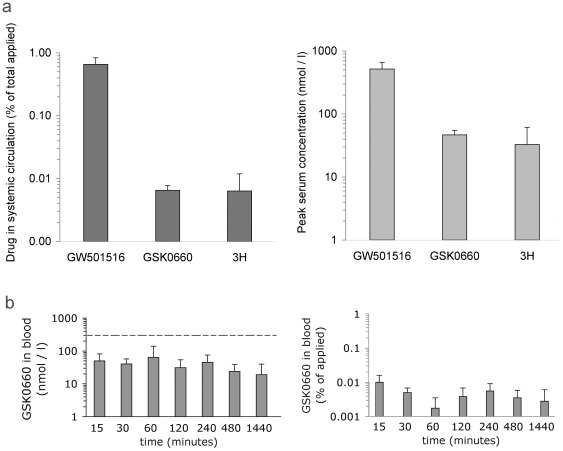
Low systemic absorption of topically applied PPAR β/δ antagonists. **A**. Peak blood concentrations of PPAR β/δ agonist GW501516, and antagonists GSK0660 and compound 3 h, respectively, at 1 h after topical application to skin. Left: Amount of drugs detected in systemic circulation, expressed as fraction of total amount applied, was calculated as detailed in methods. Right: Drug concentration expressed as molar concentration. **B**. GSK0660 concentration in blood (left) and total amount of circulating drug as fraction of amount applied (right, calculated as described in Methods) at the indicated time points after drug application. The horizontal dashed line represents the reported IC50 for GSK0660 acting on PPAR β/δ reported previously (300 nmol/L). Data shown represent average ± s.d. of n = 3 mice per data point.

**Figure 3:**
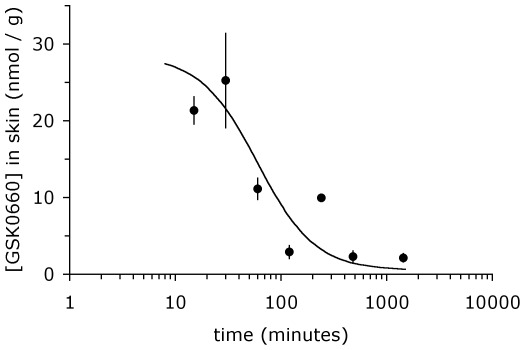
Half – life of GSK0660 after topical application to skin. 42 mg of GSK0660 ointment was applied to dorsal skin of C57Bl/6j wild type mice. Mice were sacrificed at the time after drug application indicated in the figure and drug concentration determined by mass spectrometry, as detailed in Methods. Data shown represent average ± s.d. of n = 3 mice per data point.

Targeting PPAR β/δ is potentially fraught with serious adverse effects, since PPAR β/δ impacts on a wide variety of metabolic processes. We developed a robust quantitative assay based on ULPC/mass spectrometry to allow quantification of GSK0660, as well as compound 3 h, in skin samples subjected to ointment treatment (see Methods). Using this technology, we investigated whether the topical application of PPAR β/δ antagonists to murine skin results in significant systemic drug accumulation. As shown in figure 2, peak systemic concentration of GSK0660, measured 1 h after topical application, remained well below reported EC50 and IC50 values while that of compound 3 h was slightly above the in vitro determined EC50 value at this time point (figure 1). The total amount of detectable compound was less than 0.01% of total drug applied. By contrast, the PPAR β/δ agonist used in this study, GW501516, exhibited 100-fold higher systemic absorption, achieving peak serum concentrations of 400 nM at 1 h post application, despite being concentrated 10-fold less (0.1% ointment). This concentration of GW501516 is well within the range of its expected pharmacological activity [20]. Pharmacokinetic measurements of GSK0660 in blood for 24 h after drug application showed no evidence of drug accumulation. Systemic concentration remained well below the predictive active concentration, and amounted to less than 0.01% of total drug applied being detectable (figure 2b). By contrast, the same amount of drug was able to achieve a high local concentration in skin, exhibiting a half life of approximately. 90 min (figure 3). These data demonstrate that topical administration of PPAR β/δ antagonists achieves skin specific drug application, thereby avoiding potential hazards associated with PPAR β/δ inhibition in other organ systems. Of note, since application of even 10-fold less concentrated *agonist* ointment (GW501516) achieves significant serum levels, only partial biological activity would be expected for the antagonists in this *in vivo* model.

### Lack of inflammatory changes in skin after topical application of PPAR β/δ antagonists

**Figure 4:**
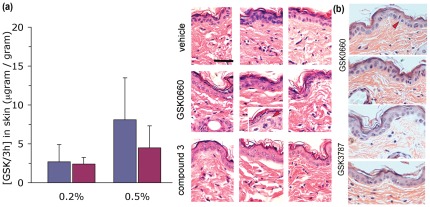
Absence of inflammatory changes induced by PPAR β/δ antagonists in skin after topical application. (a) C57Bl/6j wild type mice were treated with ointments containing GSK0660 or compound 3 h applied twice daily to shaved dorsal skin for one week. Mice were sacrificed 1 h after the last ointment application and skin tissue processed for H&E based histology and mass spectrometry, as described in Methods. Data shown represent average ± s.d. of n = 3 mice per data point (left) treated with GSK (blue columns) or compound 3 h (red). Representative histology sections of all treated mice are shown on right. The inset in the middle panel shows a section of GSK0660-treated epidermis showing apoptotic looking cells (marked by red arrow head). Horizontal bar represents 5 µm. (b) Representative H&E sections of C57Bl/6j wild type mice treated for one week with either GSK0660 (top) or GSK3787 (bottom). Red arrow-heads denoting apoptotic looking cells.

We next determined the local concentrations in skin at steady state after prolonged topical application. GSK0660 or compound 3 h were administered to shaven dorsal skin twice daily for seven days. Skin samples were then extracted and analysed by mass spectrometry for concentration determination as well as processed for histology. As shown in figure 4a, both compounds achieved high local concentrations even for the lower dose used (0.2%), suggesting active concentrations are present locally for prolonged time periods at the twice-daily dosing regimen, assuming a half life of approximately. 90 min (cf. fig. 2b). Under these conditions, neither antagonist produced notable epidermal hyperplasia (fig. 4, panels on right). The number of dermal nuclei counted per high power field was also unchanged as compared to vehicle-only treated skin (not shown). This was also found for the alternative PPAR β/δ antagonist GSK3787 (figure 4b). Infrequently, we noted apoptotic epidermal keratinocytes (inset, middle row of H&E sections). Since these cells were also found in transgenic PPAR β/δ mice treated with the agonist only (figure S1), the exact underlying cause is unclear but unlikely to underlie the treatment effect of the antagonist creams.

### Inhibition of PPAR β/δ mediated psoriasis-like skin disease

**Figure 5:**
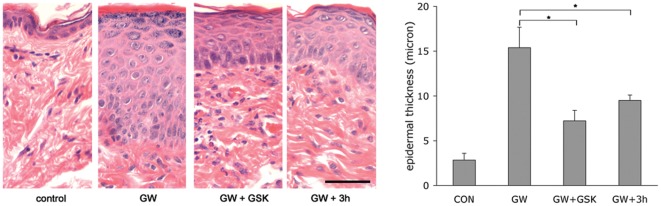
Prevention of epidermal hyperplasia by transdermal application of selective PPAR β/δ antagonists. Both the PPAR β/δ agonist GW501516 (GW) and the antagonists GSK0660 (GSK) or compound 3 h were applied topically to the skin, as described in the text. Left: representative H&E-stained paraffin-sections of dorsal skin from PPAR β/δ transgenic mice after treatment with ointments containing the indicated drugs for twenty days, as detailed in Methods. Horizontal bar represents 5 µm. Right: quantification of H&E-based epidermal thickness observed in n = 4 mice per group, performed as detailed in Methods. * p<0.05 in a two-sided independent t-test.

We next sought to determine whether skin-targeted administration of PPAR β/δ antagonists would be sufficient to inhibit PPAR β/δ agonist-driven development of skin disease. In a first set of experiments we applied both the agonist (GW501516), as well as either antagonist (GSK0660 or compound 3 h) directly to the skin in order to minimise pharmacokinetic differences associated with alternative routes of drug administration (i.e. oral verus transdermal) between the competing chemicals. GW501516 was formulated as an 0.1% ointment and applied five times per week to shaved dorsal skin of PPAR β/δ transgenic mice. This agonist concentration was chosen because (a) lower agonist concentrations had resulted in significantly prolonged time-to-onset of the phenotype in pilot experiments and (b) higher concentrations would not achieve a molar excess of the antagonist (using 1% ointments for the antagonists), which was important since the available *in vitro* data suggested that competitive antagonism at the receptor might not be achieved at equimolar concentrations of both agonist and antagonist. Antagonist-containing ointments were applied once per day six hours apart from the agonist in order to minimise any influence of penetration of both chemicals. Mice receiving GW501516 ointment and vehicle-only served as positive control, while mice receiving only vehicle ointments for both the antagonist as well as the agonist served as negative control. After 20 days of treatment, mice were sacrificed and skin samples processed for H&E histology. As shown in figure 5, both GSK0660 and compound 3 h, respectively, significantly attenuated the psoriasis – like epidermal hyperplasia induced by GW501516-mediated activation of PPAR β/δ. These data suggest that bioavailability of both antagonists is sufficient upon transdermal delivery to compete for agonist-binding to the receptor *in vivo*. MS-based quantification of GSK0660 in PPAR β/δ mice 12 h after the last cream application showed a concentration of 48 ± 18 ng/g of tissue while no GSK was detectable in blood (threshold of detection: 25 nmol/l), showing that penetration through inflamed skin with altered permeability properties does not lead to increased local or systemic accumulation of GSK0660 after prolonged administration.

**Figure 6:**
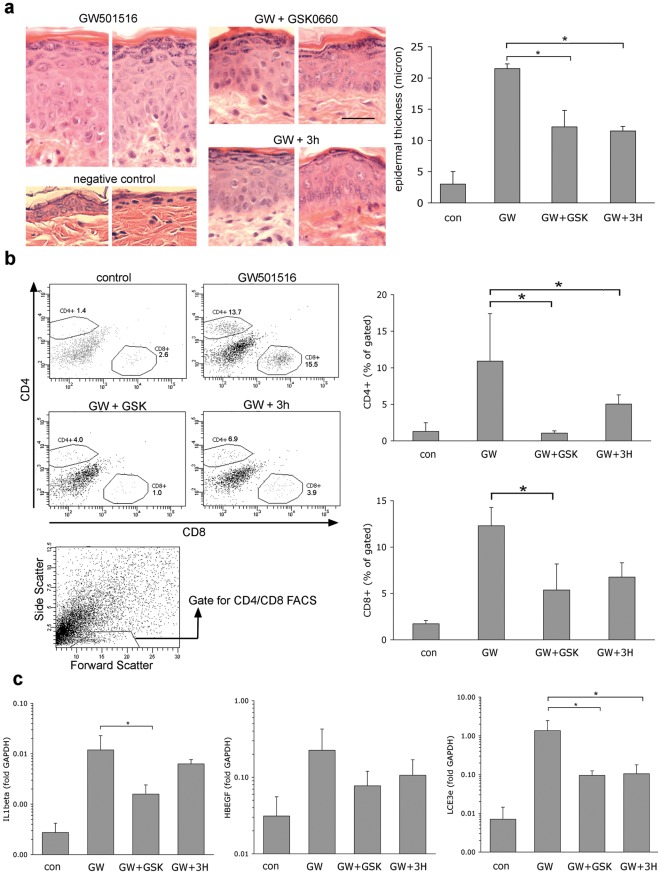
Reversal of psoriasis-like skin disease in PPAR β/δ mice by PPAR β/δ antagonists. Skin disease was induced by systemic oral administration of the PPAR β/δ agonist GW501516. (Control mice received standard chow). Subsequently, GW501516 dose was lowered to allow maintenance of phenotype as described in the text and mice were treated twice daily with either vehicle only (GW501516 group) or antagonist-containing ointments, as indicated. **A**. Reversal of epidermal hyperplasia, performed as described in the legend for figure 5. Horizontal bar represents 5 µm. **B**. Reduction of T cell infiltration. Skin samples of the treated skin regions were processed and stained for FACS analysis as described in Methods. Scatter plots shown on left show representative data, column diagrams on right show quantification of FACS data in n = 4 mice per group. The scatter plot shown at the bottom indicates the lympocyte gate used for quantification of CD4/CD8 cells, as previously described [21]. **C**. Quantification of target genes previously been shown to be induced in PPAR β/δ transgenic mice by qPCR, as detailed in Methods. * p<0.05 in a two-sided independent t-test.

We next investigated whether PPAR β/δ antagonists are able to reverse established skin disease. For this experiment, we administered the agonist GW501516 orally in order to allow twice – daily topical application of the antagonist without possible interference with drug penetration. We thus induced skin disease by oral dosing of GW501516, using a modified dosing regimen to that previously described as detailed in Methods. Three weeks after initiation of treatment with antagonists, mice were sacrificed and skin samples analysed. As shown in figure 6a, both PPAR β/δ antagonists were able to partially reverse epidermal hyperplasia. The influx of both CD4+ and CD8+ T lymphocyte subsets was also reduced, as shown in figure 6b. (cell numbers were too low to allow quantification of IL17+ T cell subsets). Finally, we also quantified expression levels of genes previously shown to be induced by PPAR β/δ in the skin [21], HB-EGF, a direct target gene of PPAR β/δ [22], as well as two strongly induced indirect target genes, IL1b and LCE3e. As shown in figure 6c, the upregulation of both HB-EGF and LCE3e was partially reversed by treatment with both PPAR β/δ antagonists, although this reached statistical significance only for LCE3e. Reversal of IL1b expression was only observed using the ointment containing GSK0660. Taken together, these data show that transdermal application of PPAR β/δ antagonists is able to reverse established psoriasis-like disease in PPAR β/δ transgenic mice.

### Reduced-frequency PPAR β/δ antagonist ointment application may be feasible using an irreversible antagonist

**Figure 7:**
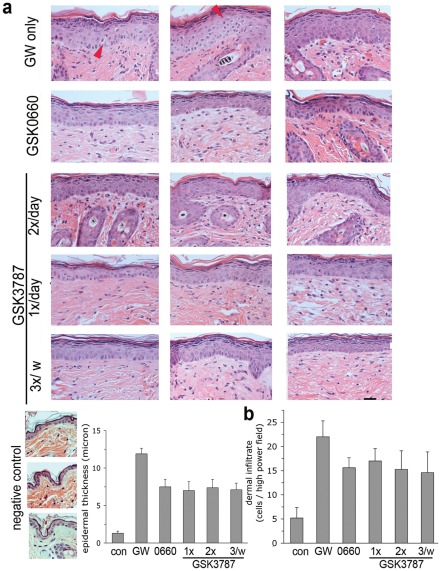
Control of PPAR β/δ – mediated skin disease using reduced-frequency application of ointment containing an irreversible PPAR β/δ antagonist. Skin disease in PPAR β/δ transgenic mice was induced by i.p. injection of the agonist GW501516. Additionally, mice were shaved on their abdomen and were treated with vehicle-ointment or ointment containing either GSK0660 (twice daily) or GSK3787 at the indicated frequencies. Red arrow denotes apoptotic cells noted in the GW-only treatment group. **A**. Top: Representative H&E stains from 3 different mice in each treatment group. Horizontal bar represents 5 µm. Bottom: Quantification of epidermal thickness (p<0.01 in all treatment groups vs. GW-only). **B**. Quantification of dermal infiltrate. Data shown represent average ± s.d. of five mice per group.

**Figure 8:**
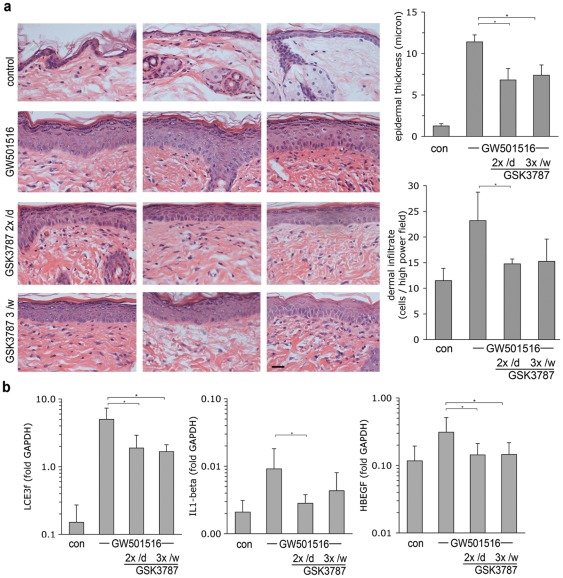
Effect of GSK3787 on PPAR β/δ -mediated skin disease applied either twice daily or three times per week (once per day). **A.** Left: representative H&E stains from 3 different mice in each treatment group. Horizontal bar represents 5 µm. Right: Quantification of epidermal thickness and dermal nuclei, respectively. * p<0.05 vs. GW501516 only. B. Q-PCR based quantification of the PPAR β/δ target genes LCE3f, IL1β, and HBEGF, as in figure 6c. Data shown represent n = 5 mice per treatment group.

The half-life of GSK0660 suggested that the frequency of cream application might be limiting for treatment efficacy. Indeed, we found that twice-daily ointment application was required for full efficacy (fig. S1). The PPAR β/δ antagonist GSK3787 has been shown to covalently bind to its target, causing permanent inactivation. Since this property may be extremely useful clinically by offering the potential of less frequent cream application, we explored the effect of GSK3787 in the present system. As shown in figure 7, treatment using GSK3787-containing ointment proved to be as effective as GSK0660 in preventing epidermal hyperplasia (fig. 7a), as well as reducing the amount of dermal infiltrate (fig. 7b), even when applied only 3× per week. The tissue level of unmodified GSK3787 in lesional skin (GSK3787 covalently attached to PPAR β/δ could not be quantified) did not differ significantly between the three treatment groups 16 h after the final application and was found overall slightly higher than that found for GSK0660 (240 ± 116 nmol/g of tissue vs. 91 ± 21 nmol/g for GSK0660), indicating that slightly better tissue penetration may contribute to treatment efficacy. Efficacy of GSK3787 at reduced frequency application was further confirmed in an additional experiment testing the effect of twice-daily versus three times weekly application of GSK3787 (figure 8). This experiment also verified suppression of the PPAR β/δ target genes LCE3f, IL1-beta, and HBEGF (fig. 8b). Quantification of GSK3787 in blood of at the end of the experiment (16 h after last cream application) yielded a concentration of 445 ± 429 nmol/l, suggesting higher systemic resorption than GSK0660. Systemic resorption appears to be facilitated through inflamed skin since GSK3787 blood concentration in healthy mice after 20 days of twice-daily treatment was only 50.2 ± 25.7 nmol/l (n = 3 mice). Nonetheless, the therapeutic activity is mediated locally, since efficacy was limited to the area of cream application (figure S2). Taken together, the data show that irreversible covalent modification of PPAR β/δ may harbour the potential of less frequent ointment application.

## Discussion

The treatment of psoriasis with PPAR ligands has been previously explored. Since PPAR β/δ may act as a direct antagonist to PPARγ [23] and PPARγ activation inhibits STAT3 [24], activation of PPARγ unsurprisingly has a mild inhibitory effect on psoriasis [25], [26]. Systemic administration of the PPARγ agonist pioglitazone to psoriasis arthritis patients, while showing signs of anti-arthritic acitivity, produced no marked reduction of PASI scores [27]. Topical application of the preferential PPARγ agonist rosiglitazone and the pan-PPAR agonist tetradecylthioacetic acid (TTA) showed no effect [26], possibly since concurrent activation of both PPARγ and PPARß/δ produces mutually neutralising effects (contrary to a commonly held view, rosiglitazone not only activates PPARγ but can also activate PPAR β/δ [18]). As selective PPAR β/δ antagonists have only recently become available, they have not yet been tested clinically. Our current data clearly support the notion that these may act anti-inflammatory in psoriasis.

The observation of therapeutic efficacy using three alternative PPAR β/δ antagonists confirms that the effects seen are mediated through PPAR β/δ binding rather than ideosyncratic off-target effects. The results presented here show some variation between the compounds tested (figs 5, 6b,c, 7, 8). GSK3787 stands out both by allowing reduced frequency application, as well as by exhbiting appreciable systemic concentrations in blood after prolonged application. More detailed kinetic follow-up studies on human skin will be required to ascertain whether this finding represents a potential safety issue. In this regard, the establishment of an ultra-sensitive quantitative mass-spectrometry assay will be instrumental.

The effect of the PPAR β/δ antagonists on the phenotype in the present model were only partial. Thus, it is possible that alternative PPAR β/δ antagonists would be more potent. More likely, however, limited efficacy is inherent to the model used here, as the antagonists need to compete with the highly potent agonist GW501516 to induce and maintain the phenotype. In psoriasis patients, such a synthetic ligand is not at work. Conversely, it is worth pointing out that oral administration of GW501516, currently explored to treat metabolic syndrome, may trigger psoriasis flares in susceptible individuals. Endogenous activation of PPAR β/δ occurs by unknown natural ligand(s). A variety of arachidonic acid species have been described as candidates. One very plausible candidate would be 12-(R)-HETE which accumulates in psoriasis [28], particularly because the enzyme catalysing its synthesis is also selectively upregulated in psoriasis [29] and localised to the same sub-epidermal compartment as PPAR β/δ, i.e. the upper spinous layer. However, none of the naturally occurring PPAR β/δ ligands display a potency comparable to GW501516. Thus, it is quite possible that application of PPAR β/δ antagonists may be more efficient than in the transgenic model.

The role of PPAR β/δ in inflammation is complex. On the one hand, some reports suggest anti-inflammatory properties (e.g. [30]). However, PPAR β/δ deficient mice do not show phenotypes indicative of increased systemic inflammatory signalling. On the other hand, PPAR β/δ activation causes pro-inflammatory changes in a number of systems, including IL-8 and IL1β induction in macrophages [18], and massive inflammatory changes in gastric tumors caused by PPAR β/δ activation, including IL1, IL6, IL24 induction [31]. When activated in the epidermis in mice, PPAR β/δ also induces the complete IL-1 signalling “module” characteristic of psoriasis including pro-inflammatory (IL1β, IL1F8), as well as anti-inflammatory cytokines (IL-1F5, IL1RA). As noted by others, the effect of PPAR β/δ activation on inflammatory signalling appears dependent on the tissue studied [31]. In the epidermis of PPAR β/δ transgenic mice harbouring PPAR β/δ expression comparable to human epidermis, it clearly acts pro-inflammatory [4]. The present data provide no evidence for pro-inflammatory changes caused by PPAR β/δ inhibition, confirming a previous report that failed to identify inflammatory changes upon systemic administration of a PPAR β/δ antagonist *in vivo* [16].

The obvious limitation of the current results is that only one model is being tested. Although widening the scope two alternative models may be desirable, this is hampered by practical considerations. The SCID model, hailed as “gold standard” by many authors, has inherent limitations: first, each data point generated requires a patient biopsy, second, biopsy grafts are fragile and not robust enough to withstand the mechanical challenge of cream treatment. It may be feasible to use this model at least to test systemic application of PPAR β/δ antagonist which is currently being explored. Of many other models propagated [32], all are limited in their modelling capacity of psoriasis for a variety of reasons [33], thereby curtailing predictive power for the human system. In addition to psoriasis-specific limitations, murine skin harbours penetration properties quite different from human skin. Thus, further preclinical testing will benefit most from porcine skin for penetration aspects as well as preclinical testing of GMP-grade products on human skin. Thus, short term ex-vivo treatment of human skin obtained from surgical procedures can be used not only to further investigative the presence of keratinocyte apoptosis but also the formation of PPAR β/δ -GSK3787 adducts.

In conclusion, we here show that selective antagonists of PPAR β/δ can be transdermally delivered, achieve pharmacologically active concentrations, and are able to antagonise PPAR β/δ activation by a potent agonist delivered orally, thereby partially inhibiting the development of an inflammatory skin disease. Their potential to treat psoriasis or related conditions remains to be explored in clinical trials.

## Methods

### Ointment formulation

All PPAR β/δ antagonists were custom-synthesized by AF-Chempharm (UK). Purity was >98% (GSK0660) and 97% (3 h, GSK3787), respectively, as determined by mass spectrometry. GSK0660 and alpha-tocopherol were predissolved in DMSO, vortexed, and incorporated into Hydromol ointment at room temperature using a melamin bowl and pestle to yield final concentrations of 0.5% (w/w) GSK0660, 0.1% (w/w) alpha-tocopherol, and 5% (w/w) of DMSO, respectively. Compound 3 h was pre-dissolved in DMSO, left at room temperature for 20 min at low agitation and then incorporated into Hydromol ointment using a melamin bowl to yield final concentrations of 0.5% (w/w) 3 h, and 5% (w/w) of DMSO, respectively. GSK3787 ointment (containing 0.5% final w/w) was made exactly analogous to GSK0660 ointment. Vehicle-only contro ointment was prepared exactly as GSK0660 ointment while omitting the chemical itself. Ointments were stored in airtight containers at 4°C in the dark and made fresh once a week.

### Chow formulation

All GW chow concentrations were made up by diluting a stock of powdered chow containing 0.3% GW501516 with standard powdered chow and by mixing thoroughly. GW chow was stored at 4°C and replaced approx. once a week.

### Evaluation of peak serum concentration and GSK0660 pharmacokinetics

GSK0660 ointment was prepared fresh, loaded into a 1 mL syringe allowing variability of drug to remain less than 10% CV, and 42 ± 5 mg of ointment was applied to 2×2 cm of shaven dorsal skin of C57Bl/6j wild type mice. Mice were sacrificed 1 h post application in a CO_2_ chamber (for peak blood concentrations determined for GW501516, GSK0660, compound 3 h), or at the time points indicated in the figure. 50 µL of cardiac blood were diluted 1∶2 (v∶v) with milipore water and snap frozen immediately. Previously treated skin segments were shaved, tape-stripped three times to remove residual hair and non-absorbed ointment on the surface, and then snap frozen in liquid nitrogen. Frozen skin was ground and ten times the volume of methanol-water (1∶1 v/v) was added per weight unit of skin. A minimum of 20 mg of skin was used per each sample. Samples were homogenized with a Covaris S2 acoustic homogeniser. For both cardiac blood and skin homogenate, proteins were precipitated by adding 3 volumes of acetonitrile containing a suitable internal standard. All samples were then centrifuged for 10 minutes at 2800 rpm before transfer of 200 µL of supernatant into a 96 well plate and 100 µL of milliQ water added. Calibration curves were constructed in either untreated homogenised skin or blood to cover at least 3 orders of magnitude (1–2000 ng/mL) and extracted as for aforementioned study samples. All cardiac blood, skin samples and standards were then investigated by mass spectrometry as described below. For calculation of total blood volume, mice were weighed and an average blood volume of 7% (mL/g) of body mass was assumed.

### UPLC and mass spectrometry

An Acquity ultra high performance liquid chromatography (UPLC) system; consisting of an autosampler, the Acquity UPLC Sample Manager with Sample Organiser, a pump, the Acquity UPLC Binary Solvent Manager, an in-built column oven, a UV detector, the Acquity UPLC Photo Diode Array) and a Quattro Premier XE triple quadrupole mass spectrometer (Waters, UK). Masslynx version.4.1 data acquisition software, was used in the determination of GW501516, GSK0660, compound 3 h, and GSK3787 in blood and skin samples using positive electrospray ionization. The analytical column was an Acquity UPLC BEH C18, 2.1 mm i.d. ×50 mm length, 1.7 µm particle size (Waters, UK). No guard column was used, but a vanguard 0.2°C.

Mobile phase A was Milli-Q reverse phase de-ionised water with formic acid (0.1%, v∶v), mobile phase B was acetonitrile with formic acid (0.1%, v∶v). Mobile phase was delivered at a flow rate of 0.6 mL/min. Gradient elution was used, for MRM analysis mobile phase B was set initially at 5% for 0.5 min was then linearily increased to 95% over 1.5 min and kept at 95% for 0.5 min. Finally mobile phase B was returned to 5% over 0.1 min and maintained at this composition for 0.4 min. Total run time 3 min.

The entire flow was introduced into the Mass spectrometer. The autosampler was set at 4°C and an injection voloume of 5 µL was used (partial loop). Mass spectrometric detection was performed using Multiple reaction monitoring (MRM) using the following mass transitions; GW501516, *m/z* 454.21 to 257.13, cone voltage (CV) 15 V, collision energy (CE) 33 V; GSK0660, m/z 419.20 to 214.17, (CV) 25 V, (CE) 19 V; compound 3 h, m/z 504.33 to 297.19 (CV) 5 V, (CE) 12 V. Desolvation temperature was 500°C and gas flow 1000 L/h (Nitrogen). An in-house proprietary internal standard was used for determination of all 3 analytes. Example chromatograms of standard, QC, Sample and blank are presented as well as the calibration curves are shown for GW501516 (Methods S1), as well as GSK0660 and compound 3H (Methods S2).

### Animal handling and dosing

All animal studies reported here were subject to approval of Tayside Research Ethics Committee, and are conducted in accordance with UK Home Office procedures (project licence 60/3800, licence holder JF). Breeding of PPAR β/δ transgenic mice and genotyping were all carried out as described previously [21]. For the experiments described in Figure 6, GW501516 was administered at a concentration 0.002% in powder chow for 48 h to induce transgene induction, followed by reducing concentration to 0.0025%. Application of antagonists in ointment was initiated after 20 days and continued twice daily for 3 weeks. For the experiment described in figure 7, skin disease was induced by 3× weekly i.p. injection of 150 µg GW501516 dissolved in 5% DMSO/PEG 700 after additional pilot studies had shown that this route of dosing allowed for tighter control of GW501516 serum concentration, reduced inter-individual variability in phenotype severity, as well as reduced overall consumption of GW501516.

### Quantification of acanthosis

Mice were sacrificed, skin samples obtained and processed for H&E based histology as described [21]. All samples were photographed at 200× magnification. Epidermal thickness was measured for all slides by a blinded observer at perpendicular angle to the basement membrane. The absence of variable planes of tissue sectioning as a confounder for apparent epidermal thickness was ruled out by verifying visible perpendicular insertion of hair follicles into the epidermis. For each sample, two measurements were taken in two separately photographed non-adjacent sections of the H&E stained skin sample.

### Flow-cytometry of skin-resident T cells

Skin was shaven and cut to appr. 10–15 mm^3^ size using scissors. Skin-associated fatty and vascular tissue was thoroughly scraped off using a scalpel to reduce the presence of T cells located in blood and lymphatic vessels. Samples were incubated for 30 min at 37°C in 2 mL of RPMI incl. Pen/Strep and 10% FCS, 2 mg/mL collagenase IV (Roche, cat-nr. 110880855001) and 1.1 U/mL dispase I (Roche, cat-nr. 04942086001) reconstituted in HBSS. Skin was washed with PBS over a cell strainer (100 µm Falcon) and subsequently incubated in 2 mL of RPMI incl. Pen/Strep and 10% FCS, 0.5 µg/mL PMA, 0.5 µg/mL ionomycin for 2 h. In modification of the previously described procedure 0.61 U/µL of DNase (Invitrogen) was to minimise clumping of cells. This addition significantly increased the yield of cells. The following antibodies served for surface staining: CD4-FITC (BD, Clone RM4-4), CD8-PerCP-Cy5.5b (BD, 53–6.7). Flow cytometry analysis was performed using a BD LSR Fortessa flow cytometer and data was analysed by means of BD FACSDiva Software.

### Real-time PCR

Skin was processed by removing subcutaneous tissue and small skin pieces were snap frozen in liquid nitrogen. Frozen skin was ground and RNA was extracted using the NucleoSpin RNA/Protein kit from Macherey-Nagel. cDNA synthesis was done with the SuperScript Vilo cDNA synthesis kit from Invitrogen. Gene expression levels were quantified using TaqMan-based real-time PCR using Assays-on-Demand kits obtained from Applied biosystems according to the manufacturer's instruction and quantification performed as calibrated to the internal housekeeping gene GAPDH (LCE3f: Mm02605425, Il1β: Mm01336189, Hb-EGF: Mm00439305).

## Supporting Information

Methods S1 Example chromatograms of standard, QC, Sample and blank are presented as well as the calibration curves for GW501516.
(DOC)

Methods S2 Example chromatograms of standard, QC, Sample and blank are presented as well as the calibration curves for GSK0660 and compound 3H.
(DOC)

Figure S1 Inhibition of PPAR β/δ -induced skin disease by topically administered antagonist GSK0660 requires twice-daily application for full efficacy. PPAR β/δ – transgenic mice were dosed with GW501516 by 3× weekly i.p. injection of the agonist GW501516 as detailed in Methods, and additionally treated with vehicle or GSK0660 ointment once or twice daily. (a) Representative H&E stains for each of the treatment groups (left) as well as quantification of acanthosis, as well as dermal infiltrate (cells per high power field, right). Red arrow head denotes apoptotic cell observed in the GW-only group. (b) Expression analysis of target genes known to be induced in lesional skin of PPAR β/δ mice, as analysed by qPCR. Data shown represent average ± s.d. of four individual mice per group. * p<0.01 in a two-sided t-test.
(TIF)

Figure S2 Limited range of PPAR β/δ antagonist ointment activity. H&E samples from the abdominal area treated with antagonist ointment were obtained in the experiment described in figure 7 and images obtained from the treated area (left) as well as the edge of the treated area (right), indicating that the effect of treatment is limited to the area treated.
(TIF)
