# Supplementary material for: Skin-Targeted Inhibition of PPAR β/δ by Selective Antagonists to Treat PPAR β/δ – Mediated Psoriasis-Like Skin Disease In Vivo
Source: PLoS One. 2012 May 14;7(5):e37097. doi: 10.1371/journal.pone.0037097 (PMC3351437; doi:10.1371/journal.pone.0037097)
Supplement: Methods S1 — Example chromatograms of standard, QC, Sample and blank are presented as well as the calibration curves for GW501516. (DOC) [file pone.0037097.s001.doc]

Mass spectrometry of GW501516

Calibration curves

Sample chromatograms
