# Supplementary material for: Skin-Targeted Inhibition of PPAR β/δ by Selective Antagonists to Treat PPAR β/δ – Mediated Psoriasis-Like Skin Disease In Vivo
Source: PLoS One. 2012 May 14;7(5):e37097. doi: 10.1371/journal.pone.0037097 (PMC3351437; doi:10.1371/journal.pone.0037097)
Supplement: Methods S2 — Example chromatograms of standard, QC, Sample and blank are presented as well as the calibration curves for GSK0660 and compound 3H. (DOC) [file pone.0037097.s002.doc]

Mass spectrometry detection of GSK0660 and compound 3H

Sample chromatograms:
