# Supplementary figures and images for: Skin-Targeted Inhibition of PPAR β/δ by Selective Antagonists to Treat PPAR β/δ – Mediated Psoriasis-Like Skin Disease In Vivo
Source: PLoS One. 2012 May 14;7(5):e37097. doi: 10.1371/journal.pone.0037097 (PMC3351437; doi:10.1371/journal.pone.0037097)

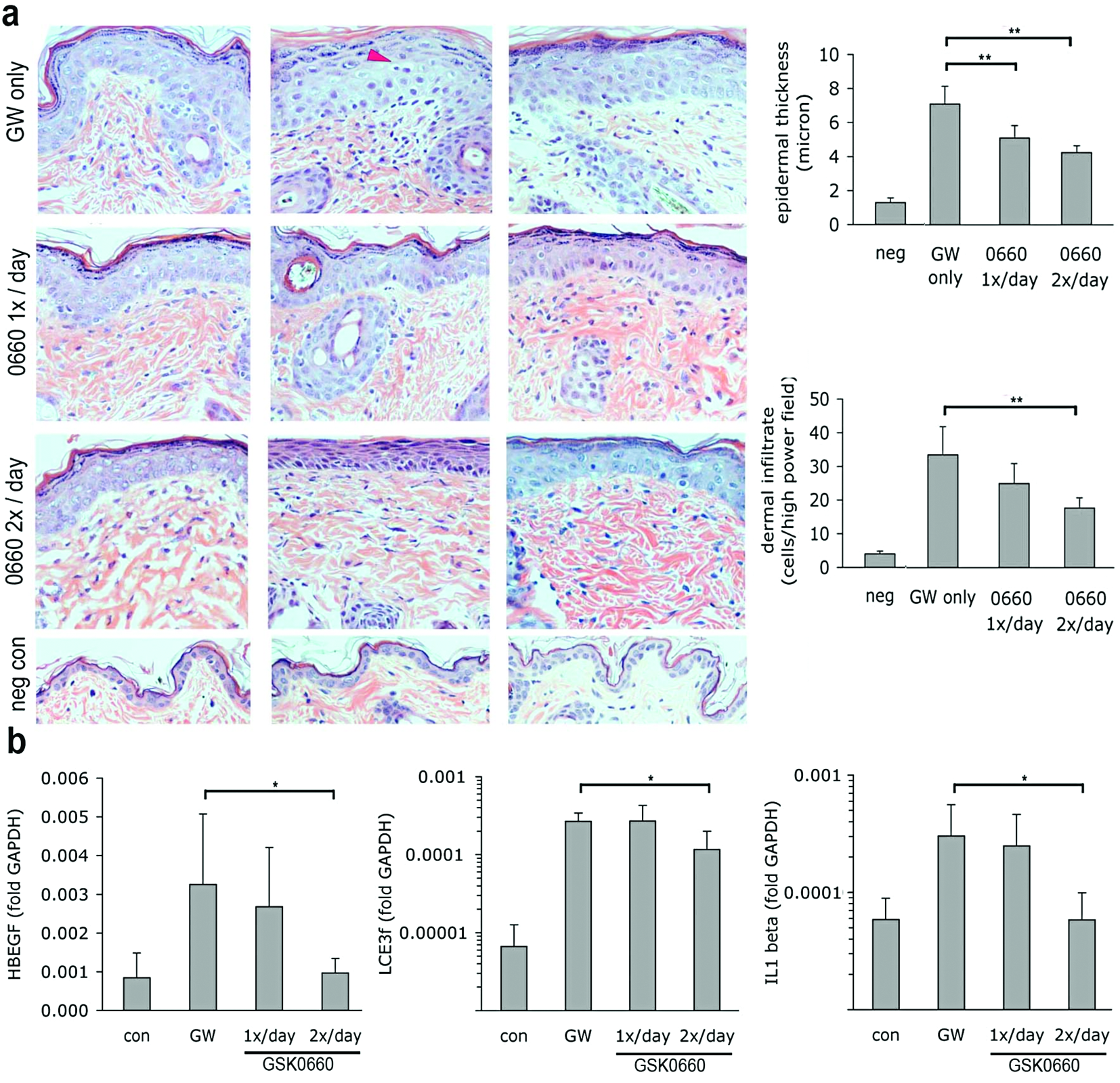

Supplement: Figure S1 — Inhibition of PPAR β/δ -induced skin disease by topically administered antagonist GSK0660 requires twice-daily application for full efficacy. PPAR β/δ – transgenic mice were dosed with GW501516 by 3× weekly i.p. injection of the agonist GW501516 as detailed in Methods, and additionally treated with vehicle or GSK0660 ointment once or twice daily. (a) Representative H&E stains for each of the treatment groups (left) as well as quantification of acanthosis, as well as dermal infiltrate (cells per high power field, right). Red arrow head denotes apoptotic cell observed in the GW-only group. (b) Expression analysis of target genes known to be induced in lesional skin of PPAR β/δ mice, as analysed by qPCR. Data shown represent average ± s.d. of four individual mice per group. * p<0.01 in a two-sided t-test. (TIF) [file pone.0037097.s003.tif]

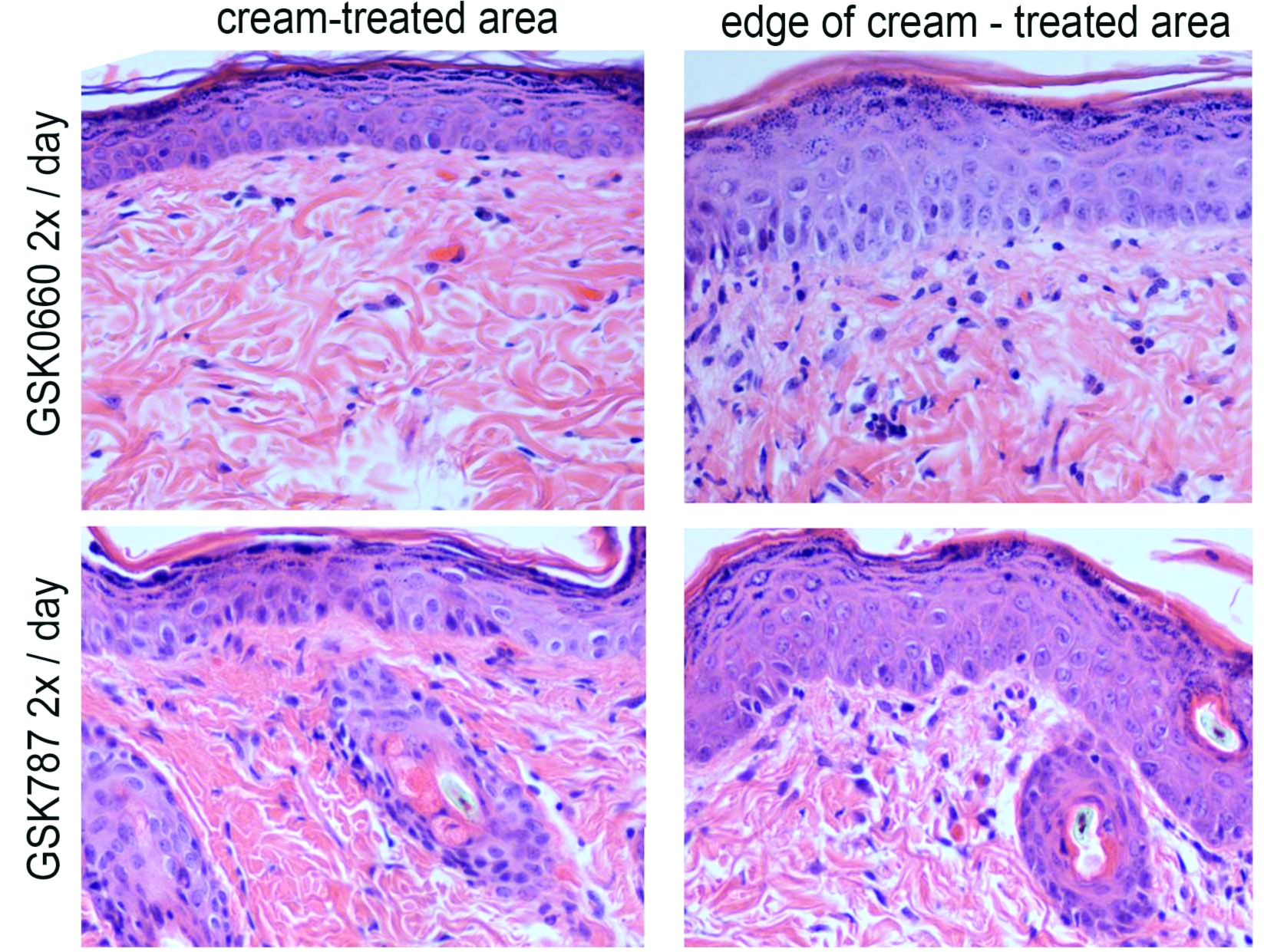

Supplement: Figure S2 — Limited range of PPAR β/δ antagonist ointment activity. H&E samples from the abdominal area treated with antagonist ointment were obtained in the experiment described in figure 7 and images obtained from the treated area (left) as well as the edge of the treated area (right), indicating that the effect of treatment is limited to the area treated. (TIF) [file pone.0037097.s004.tif]
